# Supplementary material for: The Discovery, Enzymatic Characterization and Functional Analysis of a Newly Isolated Chitinase from Marine-Derived Fungus Aspergillus fumigatus df347
Source: Mar Drugs. 2022 Aug 15;20(8):520. doi: 10.3390/md20080520 (PMC9410337; doi:10.3390/md20080520)
Supplement: Supplementary file 1 [file marinedrugs-20-00520-s001.zip › marinedrugs-1833157-supplementary.pdf]

The Discovery, Enzymatic Characterization and Functional Analysis of a Newly Isolated Chitinase from Marine-Derived Fungus *Aspergillus fumigatus* df347

Ya-Li Wu [1,2,†], Sheng Wang [3,†], Deng-Feng Yang [2], Li-Yan Yang [2], Qing-Yan Wang [2], Jun Yu [2,4], Nan Li [1,*] and Li-Xia Pan [2,*]

[1] College of Life Science and Technology, Guangxi University, Nanning 530004, China
[2] State Key Laboratory of Non-Food Biomass and Enzyme Technology, Guangxi Key Laboratory of Marine Natural Products and Combinatorial Biosynthesis Chemistry, Guangxi Academy of Sciences, 98 Daling Road, Nanning 530007, China
[3] Nanning Pangbo Biological Engineering Co., Ltd., Nanning 530004, China
[4] College of Food and Quality Engineering, Nanning University, Nanning 530200, China
* Correspondence: linan97690612@163.com (N.L.); panlixia@gxas.cn (L.-X.P.); Tel.: +86-1350-7868-042 (N.L.); +86-1376-8513-581 (L.-X.P.)
† These authors contributed equally to this work.

Contents

Figure S1. Phylogenetic tree analysis of *Af*Chi28......3

Figure S2. Sequence and structural analysis of domains of *Af*Chi28.......4

Figure S3. Agarose gel verification diagram after restriction digestion......4

Figure S4. SDS-PAGE analysis of *Af*Chi28......5

Figure S5. TLC of colloidal chitin hydrolysates produced by *Af*Chi28.......5

Figure S6. The degree of polymerization of the hydrolysis products after 6 h of reaction of *Af*Chi28 with colloidal chitin were analyzed by Q Exactive LC-MS.......6

Table S1. Kinetic parameters of *AfChi28* towards colloidal chitin......7

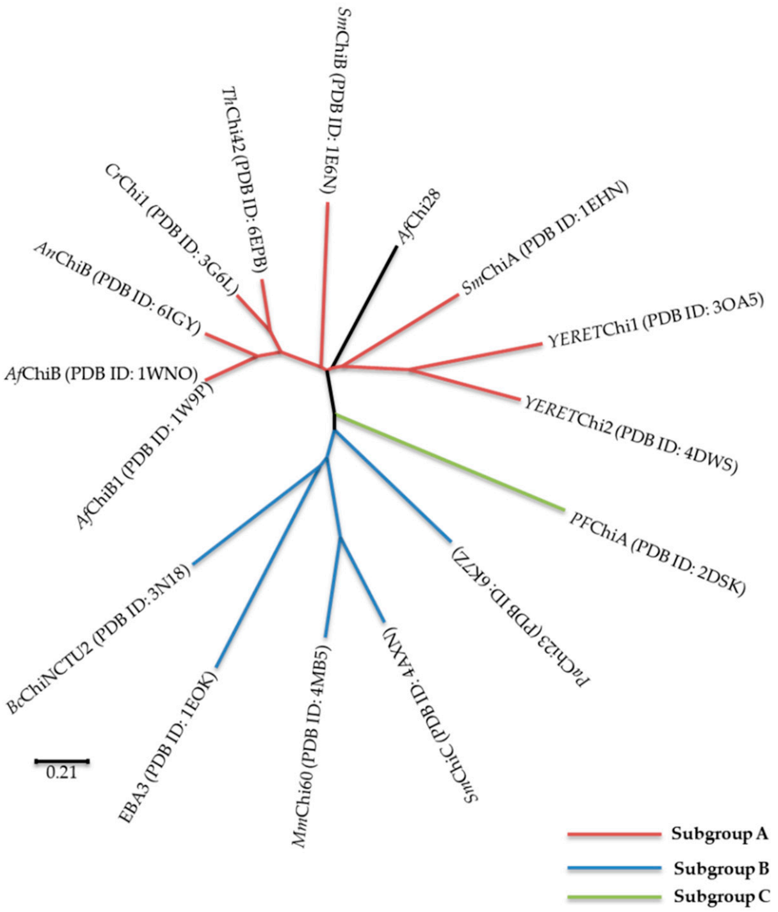

Figure S1. Phylogenetic tree analysis of *Af*Chi28 and 15 different protein sequences with high identities in Protein Data Bank. The PDB ID also show in the figure. The Gene Bank number of *Af*chi28 from NCBI database is OK 302920.

Tree scale: 0.21 substitutions per nucleotide position.

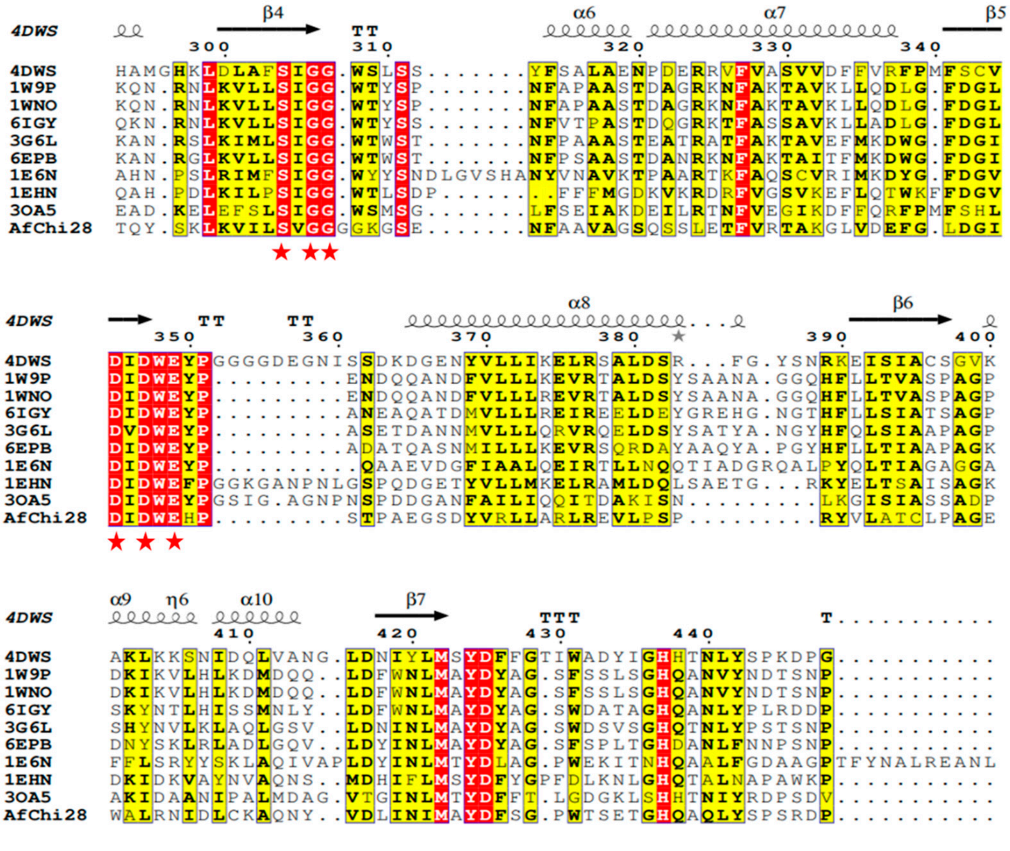

Figure S2. Sequence and structural analysis of domains of *Af*Chi28. Sequence alignment analysis of *Af*Chi28 with other subgroup A chitinases, respectively. The different sources of chitinase are indicated by their corresponding Protein Data Bank ID. The motif that was predicted to be associated with substrate specificity is indicated by red star (★).

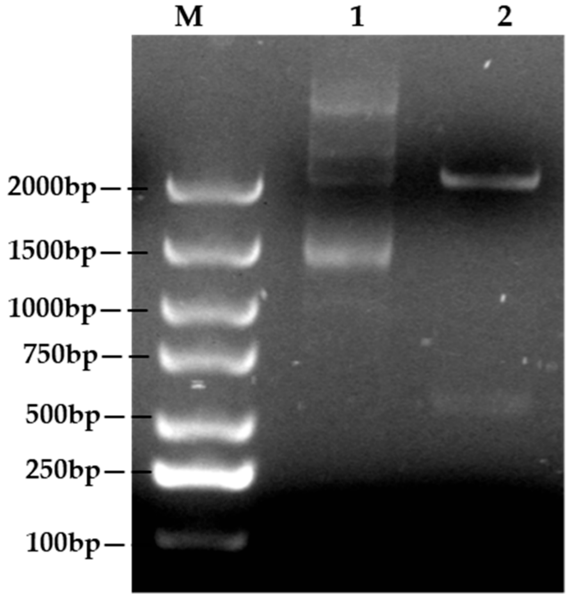

Figure S3. Agarose gel verification diagram after restriction digestion; Lane M: DNA Marker; Lane 1: pET28a (+)- *Af*chi28 (1G2800); Lane 2: Target gene.

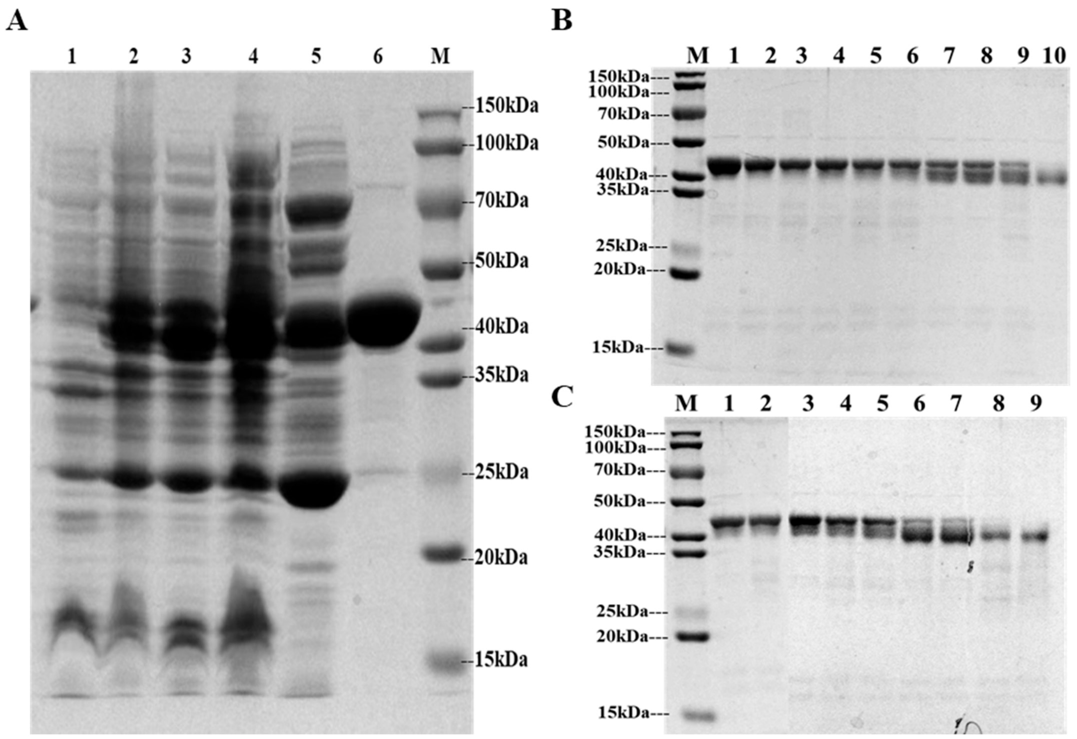

Figure S4. SDS-PAGE analysis of *Af*Chi28. Lane M: low molecular weight protein standards (150, 100, 70, 50, 40,35, 25 and 15 kDa). (A)SDS-PAGE analysis of *AfChi28* protein expression and purification. Lane 1, Cell lysates; Lane 2, The crude enzyme in the supernatant of lysates; Lane 3, the flow through; Lane 4 and 5, the flow through of impurity protein eluted by 50 mM phosphate buffer; Lane 6, purified protein eluate. (B) SDS-PAGE analysis of the stability of purified *AfChi28* protein at 30 °C incubation. Lane 1 is for the purified *AfChi28* with the concentration was 0.242 mg/ml. Lane 2 to lane 10 was supernatants collected after different incubation time (from left to right：2 h, 4 h, 6 h, 8 h, 12 h, 16 h, 24 h, 36 h and 48 h). (C) SDS-PAGE analysis of the stability of purified *AfChi28* protein at 45 °C incubation. Lane 1 to lane 9 was supernatants collected after different incubation times (from left to right：2 h, 4 h, 6 h, 8 h, 12 h, 16 h, 24 h, 36 h and 48 h).

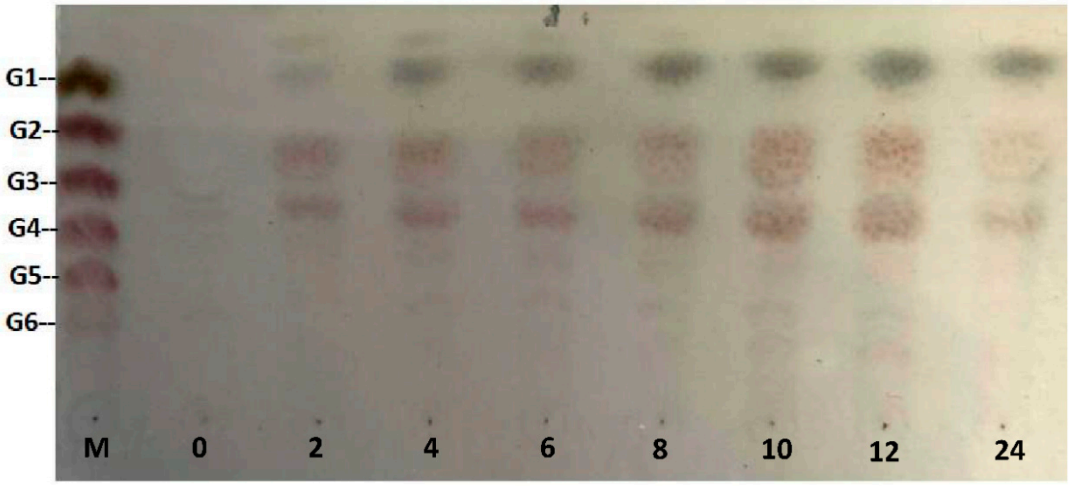

Figure S5. TLC of colloidal chitin hydrolysates produced by *Af*Chi28. Lane M, the standard of (GlcNAc)n (n = 1–6); Lane 0, control of chitin; Lane 2 to lane 24 represent the products collected at different degradation times respectively (different degradation times from left to right:2h, 4h, 6h, 8h, 10h, 12h and 24h).

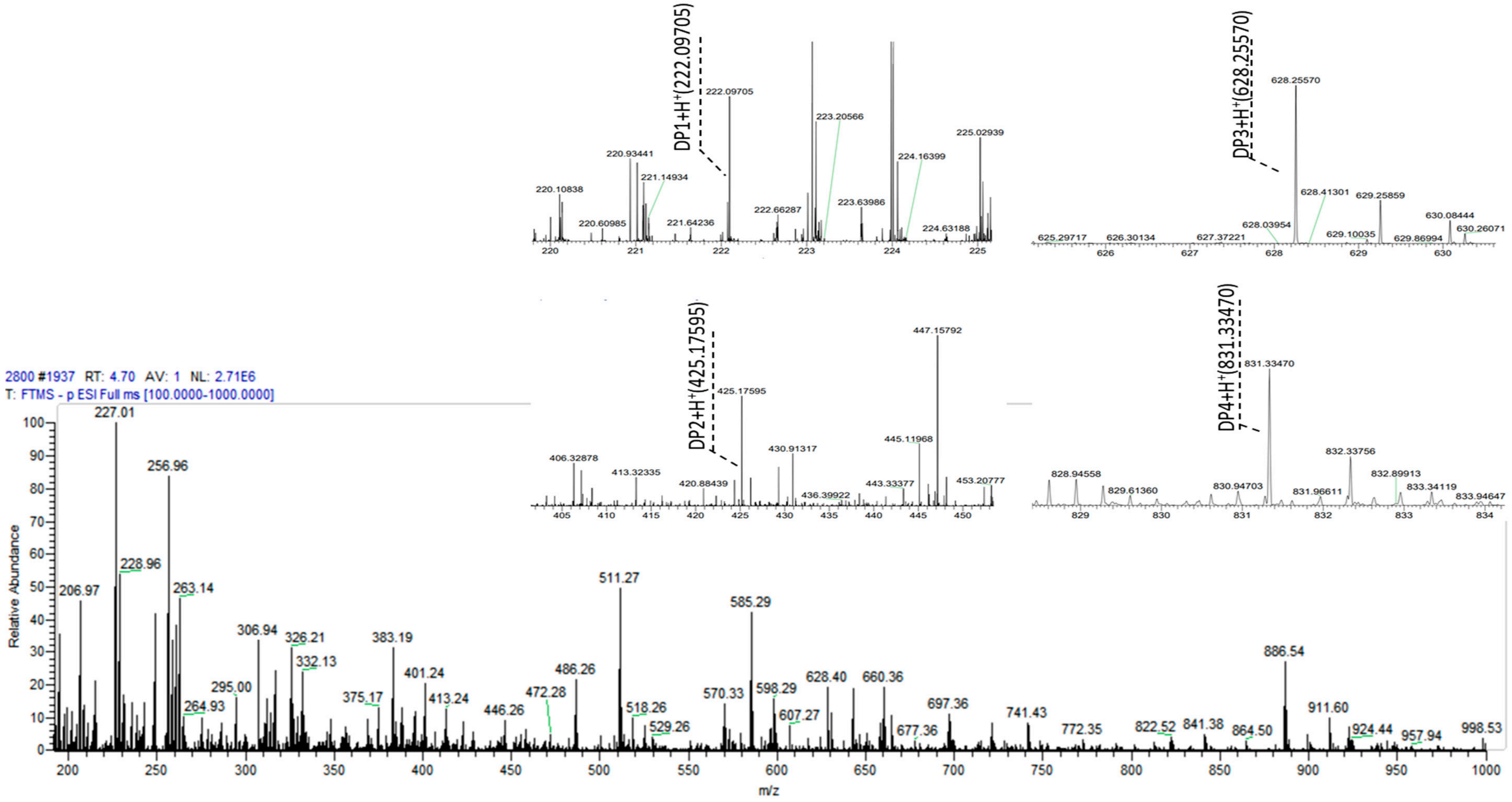

Figure S6. The degree of polymerization of the hydrolysis products after 6 h of reaction of *Af*Chi28 with colloidal chitin were analyzed by Q Exactive LC-MS.

Table S1. Kinetic parameters of *Af*Chi28 towards colloidal chitin.

Parameters	*AfChi28*
K_m(mg/mL)	2.003±0.01
K_{cat}(s^{-1})	0.0093
K_{cat}/K_m(mL/s·mg)	0.0349
